# Antioxidant-biocompatible and stable catalase-based gelatin–alginate hydrogel scaffold with thermal wound healing capability: immobilization and delivery approach

**DOI:** 10.1007/s13205-022-03131-4

**Published:** 2022-02-20

**Authors:** Heidi Mohamed Abdel-Mageed, Amira Emad Abd El Aziz, Batoul Mohamed Abdel Raouf, Saleh Ahmed Mohamed, Dina Nada

**Affiliations:** 1 Molecular Biology Department, National Research Centre, El Behoth St, Dokki, Cairo, Egypt; 2 Centre of Excellence, Arab Academy for Science and Technology and Maritime Transport, Alexandria, Egypt; 3 Pediatric Department, Faculty of Medicine, Ain Shams University, Cairo, Egypt; 4 Pharmacology and Biochemistry Department, Faculty of Pharmacy, The British University in Egypt (BUE), Cairo, Egypt

**Keywords:** Burn wound healing, Catalase immobilization, Gelatin alginate biopolymers, Half-life, Hydrogel, Stabilization, Thermal injury

## Abstract

Hydrogel-based matrix prepared using biopolymers is a new frontier of emerging platforms for enzyme immobilization for biomedical applications. Catalase (CAT) delivery can be effective in inhibiting reactive oxygen species (ROS)-mediated prolongation of the wound healing process. In this study, to improve CAT stability for effective application, gelatin(Gel)–alginate (Alg) biocompatible hydrogel (Gel–Alg), as immobilization support, was prepared using calcium chloride as an ionic cross-linker. High entrapment efficiency of 92% was obtained with 2% Gel and 1.5% Alg. Hydrogel immobilized CAT (CAT–Gel–Alg) showed a wide range of pH from 4 to 9 and temperature stability between 20 to 60 °C, compared to free CAT. CAT–Gel–Alg kinetic parameters revealed an increased *K*_m_ (24.15 mM) and a decreased *V*_max_ (1.39 µmol H_2_O_2_/mg protein min) × 10^4^. CAT–Gel–Alg retained 52% of its original activity after 20 consecutive catalytic runs and displayed improved thermal stability with a higher *t*_1/2_ value (half-life of 100.43 vs. 46 min). In addition, 85% of the initial activity was maintained after 8 weeks’ storage at 4 °C. At 24 h after thermal injury, a statistically significant difference in lesion sizes between the treated group and the control group was reported. Finally, our findings suggest that the superior CAT–Gel–Alg stability and reusability are resonant features for efficient biomedical applications, and ROS scavenging by CAT in the post-burn phase offers protection for local treatment of burned tissues with encouraging wound healing kinetics.

## Introduction

Enzymes are outstanding biocatalysts that offer several remarkable advantages such as substrate specificity, operation under mild processing conditions, and specific biocompatible by-products (Abdel-Mageed et al. 2021a). Enzymes offer numerous promising applications in the biotechnological, biomedical, and pharmaceutical fields. Catalase (CAT) enzyme (EC 1.11.1.6) is an oxidoreductase enzyme that enables cells to remove reactive oxygen species (ROS) specifically hydrogen peroxide (H_2_O_2_) that causes oxidative stress. CAT acts by breaking down hydrogen peroxide into water and molecular oxygen (Abdel-Mageed et al. 2012). When the concentration of ROS exceeds the cellular antioxidant defenses including ROS-scavenging enzymes, the oxidative stress is typically cytotoxic. Hence, there is a growing interest in the implications and the physiological significance of antioxidant CAT with a special interest in the development of biomedical and biotechnological products (Wang et al. 2021). CAT could enhance the therapeutic effects against ROS-induced acute kidney injury, acute liver injury, and wound healing through ROS removal (Wang et al. 2021). The pathological role of ROS in the inflammatory phase during wound healing is a result of the induced oxidative stress (Kurahashi and Fujii 2015). Previous studies have demonstrated the beneficial effect of CAT in wound healing (Abdel-Mageed et al. 2018a; Hu et al. 2017). However, efficient delivery of enzymes for therapeutic applications is usually hampered by its intrinsic low stability under operational conditions, high sensitivity to processing and storage conditions, and short half-life (Abdel-Mageed et al. 2020).

Immobilization is a well-established method for enzyme stabilization. Immobilization involves a chemical or a physical practice in which enzymes are fixed to or enclosed to support, generating a heterogeneous immobilized enzyme matrix that resembles the enzyme natural mode in biological cells. Selecting the best-suited immobilization support and the efficient methodology are the cornerstones for a successful immobilization process (Abdel-Mageed et al. 2019a). Physical entrapment is a favorable immobilization methodology that offers a simple procedure under mild conditions, low-cost, high activity recovery, and stability. Additionally, immobilization results in enhanced thermal and operational stability, possible enzyme recovery and reusability, and improved product yield, which are favorable for industrial applications (Abdel-Mageed et al. 2018b, 2019a).

Hydrogels are a three-dimensional structured hydrophilic polymeric network made of hydrosols (water-absorbing natural such as alginate, and pectin or synthetic polymers such as polyethylene glycol and polyethylene oxide) that quickly swells in water retaining a significant amount (exceeds 90% water) within the network while preserving a semi-solid consistency. In addition, hydrogels are presented as an advantageous matrix for CAT immobilization preserving its three-dimensional structure. Hydrogels including gelatin and alginate have been disclosed as ideal physicochemical mimetic biological scaffolds that can be used as a delivery vehicle for wound healing, as they can maintain a moist environment, absorb wound exudates, and fight bacteria (Sivaraj et al. 2021; Zhang et al. 2021).

Alginate is an anionic biopolymer obtained from brown algae that are usually used for hydrogel production and has been employed in several biomedical applications, protein/drug delivery, tissue engineering, and wound healing (Aderibigbe and Buyana 2018; Miao et al. 2018). Sodium Alginate is composed of irregular blocks of *β*-d-mannuronic acid (M-block) and 1*–*4 linked *α*-l-guluronic residues (G-block) and offers distinct advantages over presently used wound dressing including improved absorption capacity of wound exudates, minimizes the risk of bacterial infections, reduces adverse allergic effects, and improves wound healing because of its biocompatibility, and non-immunogenicity. In addition, sodium alginate maintains humid surroundings that enhance reepithelization and fast granulation. It also displays hemostatic properties, which are beneficial for bleeding wounds (Zahid et al. 2021). For biomedical applications, sodium alginate offers a unique advantage where it forms a physically cross-linked hydrogel under mild conditions in the presence of divalent cations such as Ca^2+^, Fe^2+^ or Mg^2+^, accompanied by a transition from solution to gel (Aderibigbe and Buyana 2018; Pilipenko et al. 2019). Among these cations, Ca^2+^ is the most frequently used for ionic cross-linking of alginate, with calcium chloride (CaCl_2_) demonstrated as one of the best Ca^2+^ sources for this process. The cross-linking process is chiefly accomplished via the replacement of the sodium ions of G-blocks with the divalent cations such as Ca^2+^ and bending of guluronic groups to create the egg box structure (Abasalizadeh et al. 2020). Alginates reported bio-adhesive properties and antibacterial activity is probably owed to the presence of free carboxyl groups (Cattelan et al. 2020).

Gelatin is a biocompatible and biodegradable polymer that is typically obtained from porcine, bovine, or fish collagen through acid or base hydrolysis (Liu et al. 2015). Gelatin has been recognized for wound healing application, as it possesses numerous substantial properties including plasticity, film forming and cell adhesion capacity, non-immunogenicity, ability to synthesize in situ hydrogels, and tissue growth enhancement. A gelatin hydrogel is generally prepared through physical cross-linking in water at ≥ 2% w/v and ≤ 29 °C and usually dissolves at body temperature (37 °C), making it unsuitable for biomedical applications. Several cross-linking methods are used to enhance mechanical strength and provide the long-term stability of gelatin-based hydrogel. These include thermal gelation, ionic and covalent cross-linkers, Schiff base reactions, and enzyme-mediated cross-linking. Calcium ions (Ca^2+^) play a significant role in improving the mechanical properties of hydrogels with ionically cross-linked networks (Abuelezz et al. 2020; Zhang et al. 2020). The ionic interaction between sodium alginate and gelatin occurs owing to the occurrence of ionizable amino and carboxyl groups, also due to the hydrogen interactions between the amine and carboxyl group (Li et al. 2021).

The post-burn inflammatory phase is accompanied by the massive production of ROS that is implicated in the progression of the local and distant inflammatory process (Vorauer-Uhl et al. 2002). Instantly following the thermal injury, polymorphonuclear leukocytes (PMNs) invade the lesion initiating the release of huge amounts of ROS in the interstitial fluid. Unfortunately, the concentration of antioxidant enzymes in the wound fluid is very low and insufficient to remove a large amount of ROS generated during the post-injury phase. In this regard, ROS acts directly on the cell membranes lipids (Saitoh et al. 1994). The beneficial effect of systemically administered antioxidant enzymes to prevent oxidative tissue injury is usually unsuccessful and is opposed by the bad pharmacokinetic profile of systemic enzyme administration (Steiling et al. 1999). In an attempt to find alternative strategies for successful biomedical use of CAT, a topical application using hydrogel is proposed as an effective means in inhibiting ROS-mediated impaired wound healing.

Accordingly, the integration of bioactive antioxidant CAT enzyme into Gel–Alg hydrogel emerges as a promising strategy to develop an antioxidant-efficient wound dressing. In this study, the first goal is to design biocompatible support for physical immobilization of CAT with high enzyme loading capacity, and high stability and reusability using a simple and mild methodology. The second goal is the use of the novel CAT-based hydrogel as a wound dressing matrix to prevent oxidative tissue damage for wound healing applications. Two naturally occurring hydrogels, gelatin (Gel) and alginate (Alg), were used as a support matrix for the physical immobilization of the CAT enzyme. Comparison of catalytic parameters such as temperature, pH, and kinetic parameters between the immobilized and the free enzyme was evaluated. Evaluation of the protective effect of CAT against ROS-induced wound damage was investigated using the thermal injury model.

## Materials and methods

### Materials

Bovine liver catalase (CAT) (40,000–60,000 units mg^−1^ protein), hydrogen peroxide, gelatin (Type A, porcine) (MW ~ 50,000–100,000 Da), and calcium chloride of analytical grade were purchased from Sigma Chemical Co. (St. Louis, MO, USA). Alginate was obtained from Merck KGaA (Sodium alginate, MW 50,000 Da, Merck KGaA, Darmstadt, Germany).

### Preparation of Gel–Alg hydrogels

The hydrogel matrix was prepared according to the previously published methodology (Rescignano et al. 2016). Briefly, sodium alginate and gelatin were firstly dissolved, separately, in potassium phosphate buffer, pH 7.0 at the concentration of 2% (w/v) gelatin and 1.5% (w/v) sodium alginate, followed by mixing the two obtained solutions under homogenization for 2 h at 1000 rpm with a mechanical stirrer. Then, the mixture solution was immersed into 3% CaCl_2_ solution (w/v) at 30 °C for 30 min under low stirring and stored at 4 °C for 18 h to form the hydrogel matrix (Gel–Alg).

### Determination of entrapment efficiency of CAT enzyme

Physical immobilization of CAT was achieved through the entrapment technique. In brief, 200 U mL^−1^ of CAT solution was prepared in 50 mM potassium phosphate buffer, pH 7.0, and was mixed in gelatin/alginate solution and the procedure was completed as described above to obtain CAT-based hydrogel (CAT–Gel–Alg).

Entrapment % was calculated according to the following Eq. (1):

1 $${\text{Entrapment }}\left( \% \right){ = }\frac{{\text{Total activity of immobilized CAT}}}{{\text{Total initial free enzyme activity }}} \times {100}{\text{.}}$$

### Activity assay for free and immobilized CAT

CAT activity was assayed using a simple and quantitative spectrophotometric assay with H_2_O_2_ as the substrate (Beers and Sizer 1952). The activity of CAT–Gel–Alg reaction was initiated by adding aliquots to a 3 mL of 20 mM H_2_O_2_ solution prepared in 50 mM potassium phosphate buffer, pH 7.0, to obtain a final CAT concentration of 0.2–0.4 µg protein mL^−1^ in the assay reaction mixture. Then, the time course decomposition of the H_2_O_2_ solution into molecular oxygen and water catalyzed by CAT enzyme at 25 °C was followed at 240 nm using a spectrophotometer (JASCO V-530, Japan). The calculations were performed based on the H_2_O_2_ absorbance at 240 nm with the molar extinction coefficient of 43.6 M^−1^ cm^−1^. For the free CAT activity measurements, the appropriate amount of the freshly diluted CAT samples was used as described above with a CAT concentration of 0.2–0.4 µg protein mL^−1^ in the assay reaction mixture. One unit of catalase is defined as the amount of enzyme that decomposes 1.0 µmol of H_2_O_2_ into molecular oxygen and water per min under standard assay conditions. The protein concentration was determined using the Bradford method, with bovine serum albumin as a standard (Bradford 1976).

### Characterisation of Gel–Alg hydrogel

#### Water content

The hydrated sample was dried at 105 °C until constant weight and the sample water content was calculated according to the AOAC (2000) methods using Eq. (2):

2 $$W_{{\text{c}}} = \frac{{W_{{\text{o}}} - W_{1} }}{{W_{{\text{o}}} }} \times 100,$$

where *W*_o_ and *W*_1_ are the weight (g) of hydrogel before and after drying at 105 °C till constant weight, respectively.

#### Swelling behavior

The swelling behavior of the prepared hydrogel was determined by a gravimetric method as described by Zahid et al. (2021). The hydrogel samples were weighed and immersed in 100 mL of 0.1 M potassium phosphate buffer, pH 7.0, to determine the swelling behavior at 25 °C. At predetermined time intervals, the hydrogel was removed from the buffer solution by vacuum filtration and reweighed after wiping out the excess solution on the surface gently using a filter paper. The swelling ratio of the gel was calculated using Eq. (3):

3 $${\text{Swelling}}\;\% = \frac{{W_{{\text{s}}} - W_{{\text{i}}} }}{{W_{{\text{i}}} }} \times 100,$$

where *W*_s_ and *W*_i_ are the weight (g) of the hydrated swollen sample at time *t* (after intervals of 1, 2, 3, 4, 6, 8, and 12 h) and the weight (g) of the initial sample, respectively.

### Catalytic parameters for free CAT and CAT–Gel–Alg

#### Thermal stability and half-life

The thermal stability of free and immobilized CAT was determined by measuring the residual catalytic activity at two temperatures (30 and 50 °C) in potassium phosphate buffer (0.1 M, pH 7.0) for different time intervals (15, 30, 45, 60 min). Afterward, enzyme activity was measured under standard assay conditions. The % residual CAT activity was calculated as the proportion of the residual activity retained about the initial activity at 25 °C, which was considered as 100%. The first-order inactivation rate constants *K*_d_ were calculated according to the following Eq. (4):

4 $${\text{LnA}} = {\text{LnA}}_{{\text{o}}} - K_{{\text{d}}} {\text{t}},$$

where *A*_o_ is the initial enzyme activity and *A* is the residual activity after time *t* by plotting a graph of − ln *A*/*A*_0_ on the *Y*-axis against time (*t*) on the *X*-axis. The slope of the graph gives *K*_d_ of the enzyme and *t*_1/2_ is calculated using Eq. (5):

5 $${\text{t}}_{1/2}\text{=}\frac{0.693}{{\text{K}}_{\text{d}}}.$$

#### pH and temperature

The pH profiles for free CAT and CAT–Gel–Alg were carried out at different pH values under standard assay conditions using the following buffers: 50 mM sodium acetate buffer for pH values 4.0 and 5.0 and 50 mM potassium phosphate buffer for pH values from 6.0 to 9.0 at 25 °C. The catalytic reactions were initiated by the addition of the appropriate dilution of free CAT or immobilized samples to the substrate solution. Optimum pH was considered to be at 100% catalytic activity and other activities were expressed as a percentage of this optimum 100% activity. The effect of temperature on free CAT and CAT–Gel–AGL was tested by assaying the catalytic activity after 15 min incubation at temperatures ranging from 20 to 60 °C with an interval of 10 °C. The residual enzyme activity was determined under standard assay conditions.

#### Kinetic parameters

Activity assays using H_2_O_2_ were carried out for the determination of the intrinsic kinetic parameters (Michaelis–Menten constant (km)) and maximum reaction velocity (*V*_max_)) for the free CAT or CAT–Gel–AGL. Measurements were performed using different concentrations of H_2_O_2_ ranging from 4 to 40 mM. The decomposition of each concentration of H_2_O_2_ was initiated at a fixed overall CAT concentration of 0.2 µg mL^−1^. The CAT activity assay was carried out under standard assay conditions.

#### Reusability study

The prepared CAT–Gel–Alg was employed to catalyze hydrogen peroxide decomposition reaction for several consecutive runs and CAT activity assay was carried out under standard conditions. After each reaction, the hydrogel was removed from the reaction mixture by filtration technique. Then, it was washed with potassium phosphate buffer (0.1 M, pH 7) at 25 °C three times to remove any residual substrate within the hydrogel matrix (Abdel-Mageed et al. 2012). The reusability study was performed for 20 consecutive runs and the obtained relative activities were plotted against the number of cycles.

#### Storage stability

Storage stability for both free CAT and CAT–Gel–Alg were determined at different time intervals after storage for 60 days in potassium phosphate buffer (0.1 M, pH 7.0) at 4 °C.

### Experimental setup for thermal injury model

Adult male Wistar rats (250 ± 50 g) were divided into three groups (*n* = 8, total 24 animals), kept for 7 days of acclimatization and given a standard diet and water ad libitum. The experimental design was performed according to the method described by Vorauer-Uhl et al. (2001). Thermal burn was induced by exposing the depilated back skin of anesthetized animals [were anesthetized with ketamine (40 mg kg^−1^) and xylazine (20 mg Kg^−1^)], placed in a supine position inside a plastic box open. The marked skin area was exposed to hot water (rats: 95 ± 1 °C, 15 s, 15 cm^2^) causing a uniform second-degree burn. The experimental protocol was conducted in compliance with the Institutional Animal Care and Use Committee Guide 8th Edition 2011 (IACUC) National Research Centre, Cairo, Egypt, in accordance with the national and international Guide for the Care and Use of Laboratory Animals. Treatment with CAT–Gel–Alg hydrogel was performed immediately after thermal injury and twice daily for 3 days (treated group). In addition, one group was left untreated (naive group) and one was spread with the plain Gel–Alg hydrogel (control group). The size of the lesions and wound contracture were evaluated at 24, 48, and 72 h post-trauma. The size of the lesions was determined using a planimetric method that projects the lesions’ shapes onto a transparent pattern foil.

### Statistical analysis

All experiments were carried out in triplicate. The data were analyzed using one-way analysis of variance followed by the Student’s *t* test for comparisons between groups (SPSS 19.0 was used to perform statistical analysis). Significance was indicated by *p* < 0.05 in this study.

## Results and discussion

### Preparation of Gel–Alg hydrogel

Using a simple and mild methodology Gel–Alg was successfully prepared at 2% W/V gelatin and 1.5% sodium alginate. Alginate hydrogels are typically formed after cross-linking in an aqueous medium in the presence of divalent cations such as Ca^2+^ that acts as ionic cross-linking agents. The high aqueous solubility of CaCl_2_ made it one of the most commonly employed cross-linking agents in aqueous media. It is suggested that the Ca^2+^ interacts with the G-block of the biopolymer by replacing the sodium ions of the G-blocks forming the known egg box structure. On the other hand, the M-blocks of alginate exhibit weak interactions with calcium cations (Rescignano et al. 2016). The concentration of the gelling agent, mixing time, speed and temperature were optimized in preliminary studies (data not shown). These factors should be carefully chosen as they affect gel uniformity and strength.

Uniform hydrogels with improved mechanical strength are usually produced by decreasing the rate of gelation. A potassium phosphate buffer was used to slow and control the gelation rate, as the phosphate groups in the buffer compete with the –COOH group of alginate during the reaction with calcium ions. In addition, decreasing the temperature lowers the divalent cations’ reactivity, which decreases the gelation rate resulting in a highly ordered cross-linked network that consequently improves the mechanical properties of the prepared hydrogel (Abasalizadeh et al. 2020). To overcome the alginate in situ limitations, gelatin was used in conjugation to prevent a reduction in viscosity and dissolution upon application to biological tissues. In addition, calcium chloride was used as the sole crossing agent and no organic solvents were employed during the preparation of the hydrogel to preserve the fragile nature of CAT.

### Immobilization of CAT

CAT is a highly valuable antioxidant enzyme that has great potentials for biomedical applications. Physical immobilization technique through entrapment is a mild process that does not affect the enzyme structure and hence its catalytic activity. The entrapment efficiency of CAT in Gel–Alg hydrogel was found to be 92% (Table 1). It is expected that under the mild conditions employed in hydrogel preparation and the absence of organic solvents, the CAT structure in biopolymer gel is unchanged. Additionally, it has been reported before that chemical cross-linkers such as glutaraldehyde adversely affect enzyme activity (Gür et al. 2017). High entrapment efficiency upon physical immobilization has been reported by other authors using different carriers such as hydrogels (85%, Jiang and Zhang^1993^), chitosan beads (96%, Kaushal et al. 2018), and vesicles (≥ 80%, Abdel-Mageed et al. 2012). Additionally, it is worth noting that both sodium alginate and gelatin act directly as CAT stabilizers; CAT, alternatively, prevents degradation of the hydrogel either by light or by oxidation, avoiding possible chemical reactions.

**Table 1:** Immobilization of CAT into Gel–Alg hydrogel showing enzyme activity and immobilization yield

Enzyme sample	Enzyme activity (U)	Protein (mg)	Specific enzyme activity (unit mg^−1^)	Immobilization yield (%)
Free CAT	20,580	16.5	1247.27	–
CAT–Gel–Alg	18,953	14.1	1344.18	92 ± 5

Enzyme activity assay carried out as described in “Methods”. Cross-linked hydrogel matrix composed of 2% W/V gelatin and 1.5% W/V sodium alginate. Values are means ± SD (*n* = 3)

### Characterization of the hydrogels

#### Water content of the hydrogels

The hydrogel water content is a significant measure to determine water absorbing and retaining capacity. The prepared hydrogels (Gel–Alg and CAT–Gel–Alg) exhibited a high capacity to absorb and retain water with no significant difference in behavior. The water content of Gel–Alg and CAT–Gel–Alg was 95.72 and 96.1%, respectively. These results are an indication of the ability of the hydrogel to prevent the accumulation of wound fluid by absorbing the exudates for effective wound dressing (Stephen 2004; Cattelan et al. 2020).

#### Swelling behavior

**Fig. 1:**
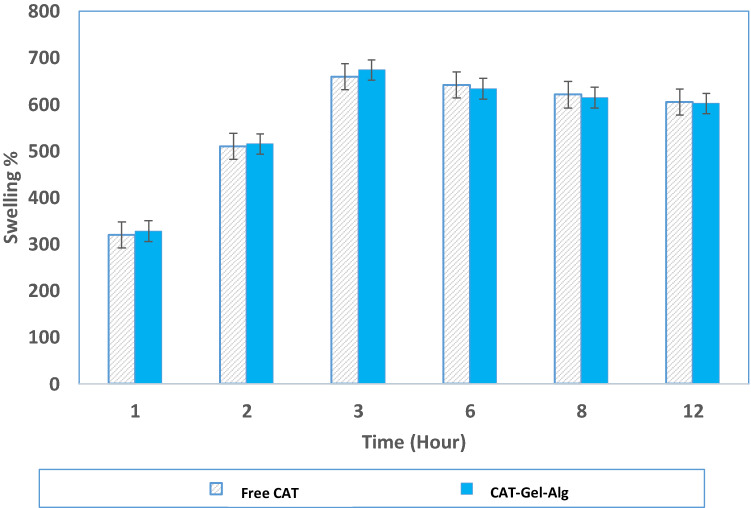
Swelling behavior of cross-linked hydrogel matrix (Gel–Alg) (2% (w/v) gelatin and 1.5% (w/v) sodium alginate) at 1, 2, 3, 4, 6, 8, and 12 h in 0.1 M potassium phosphate buffer, pH 7.0 at 25 °C. Each point represents the average of three experiments ± SD

Hydrogel swelling behavior is a primary criterion for investigating the ability to absorb water and can be utilized to verify its efficiency to protect enzyme conformational structure upon immobilization. The swelling behavior of Gel–Alg and CAT–Gel–Alg sample hydrogels as a function of time is shown in Fig. 1. Both Gel–Alg and CAT–Gel–Alg samples revealed a time-dependent swelling profile, where they started to absorb water soon after they were dropped into the deionized water with faster absorption in the first 2 h of immersion and attained a hydration equilibrium point after 3 h (Fig. 1). Gel–Alg and CAT–Gel–Alg reached a maximum absorption value of 660% and 674%, respectively, after 3 h. Similarly, Muanruksa et al. (2020) reported that the prepared alginate hydrogel absorbed a greater amount of water, as the retention time increased till an equilibrium status was reached (3 h). These results are an indication of the ability of the hydrogel to prevent the accumulation of wound fluid by absorbing the exudates for effective wound dressing.

### Catalytic parameters

#### Effect of temperature and thermal stability on CAT activity

**Fig. 2:**
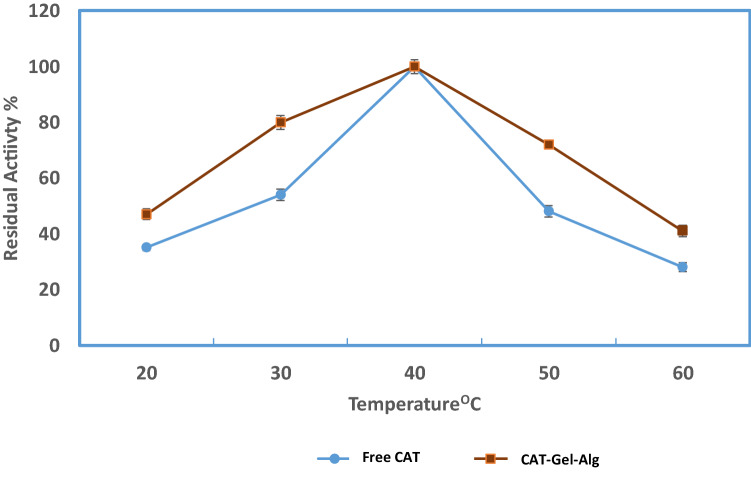
Effect of temperature on catalytic activity of free CAT and immobilized CAT (CAT–Gel–Alg) after 15 min incubation at various temperatures from 20 to 60 °C (pH 7.0: phosphate buffer, 50 mM). CAT activity assay was carried out under standard assay conditions. Each point represents the average of three experiments ± SD

Determination of thermal stability is highly essential for effective enzyme application. Figure 2 represents the thermal stability of both free and encapsulated CAT as a function of temperature. CAT activity was assayed after enzyme incubation at different temperatures ranging from 20 to 60 °C for 15 min preceding the substrate addition at pH 7.0. It is being found that both free and immobilized CAT exhibited the highest activity at 40 °C. In addition, they both showed more stability at a lower temperature between 20 and 40 °C. CAT–Gel–Alg exhibited significant stability at higher temperatures where 55% of activity was retained at 60 °C. The activity of the free CAT started to steeply decline after 40 °C and reached 28% at 60 °C. It is typically reported that immobilized enzymes exhibit improved thermal stability than the free enzyme due to the reduction of conformational flexibility in the immobilized enzyme. Similarly, Kaushal et al. (2018) and Abdel-Mageed et al. (2012) reported the improved thermal stability of CAT upon immobilization.

Table 2 shows the values of inactivation rate constants *K*_d_ as evaluated from the slope of the plot and *t*_1/2_ for free and immobilized CAT. At 50 °C the calculated *K*_d_ for free CAT and CAT–Gel–Alg was 0.015and 0.0069 min^−1^, respectively. The results show that the immobilization procedure adopted in this study improved the thermal stability of CAT as evident from *t*_1/2_ values. The *t*_1/2_ values at 50 °C demonstrate the preserving effect of immobilization on CAT activity with *t*_1/2_ values of 46 and 100 min for free CAT and CAT–Gel–Alg, respectively. Thermal inactivation and degradation of enzymes regularly take place at higher temperatures thus exhibiting lower *t*_1/2_ values (Abdel-Mageed et al. 2019b). Extensive application opportunities are expected for immobilized enzymes with improved thermal stability. Several authors have reported the improved thermal stability of CAT upon immobilization where the polymeric network serves to preserve the conformational structure of the enzyme against denaturation (Gur et al. 2017; Kaushal et al. 2018). These results also suggest that the chosen biopolymers in this study did not exert any denaturation effect on CAT even under thermal stress. This is probably because of the physical entrapment of CAT within the polymer network, the effect of the cross-linking process, and the possible protective effect of calcium in the thermostability of CAT (Arica et al. 1999).

#### Effect of pH on the CAT catalytic activity

**Fig. 3:**
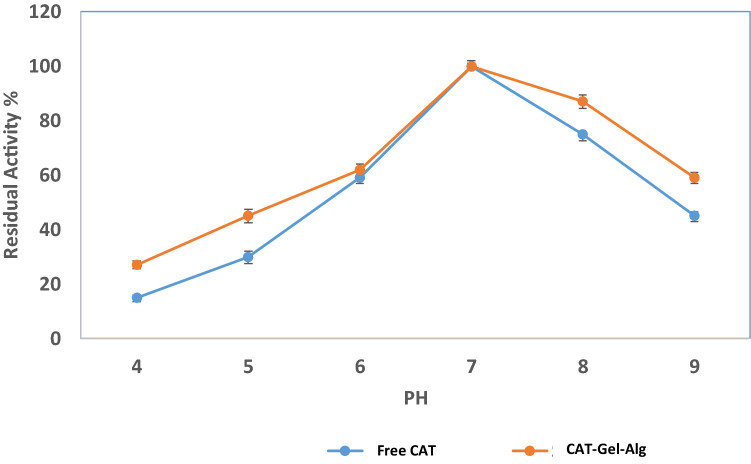
Determination of pH stability on free CAT and immobilized CAT (CAT–Gel–Alg) enzyme at various pH values pH 4.0–pH 9.0 (used buffers: 50 mM sodium acetate buffer for pH values 4.0 and 5.0 and 50 mM potassium phosphate buffer for pH values from 6.0 to 9.0) at 25. Each point represents the average of three experiments ± SD

The effect of pH on free CAT and immobilized CAT was studied to serve as an indication of the change in the chemical characterization of CAT upon immobilization. The effect of pH on enzyme activity for free CAT and CAT–Gel–Alg forms was studied with a 4–9 pH range using different buffers. Both free and immobilized CAT exhibited maximum catalytic activity at pH 7.0 using potassium phosphate buffer. At all measured pH values, CAT–Gel–Alg exhibited higher activity in comparison to the free form (Fig. 3). These improved stability results can be credited to the physicochemical stabilization of the enzyme inside the hydrogel matrix. Enzyme immobilization typically impacts the enzyme ionization state, its dissociation, and its conformational structure. Hence, an influential effect of pH is usually observed that indicates an association between the pH, stability, and the catalytic activity of the immobilized enzymes (Abdel-Mageed et al. 2019a).

**Table 2:** The first-order deactivation rate constant (*K*_d_) and half-life (*T*_1/2_) of free CAT and immobilized CAT (CAT–Gel–Alg) which were incubated at 30 and 50 °C, respectively

Temperature, °C	*K*_d_ free CAT, min^−1^	*T*_1/2_ free CAT, min	*K*_d_ immobilized CAT, min−1	*T*_1/2_ immobilized CAT, min
30	0.008	86.6 ± 9	0.0046	150 ± 18
50	0.015	46 ± 7	0.0069	100.43 ± 12

Process conditions: pH 7.0, 50 mM phosphate buffer. Values are means ± SD (*n* = 3)

#### Kinetic parameters

The velocity of an enzyme reaction is ultimately affected by the substrate concentration of that specific enzyme. This relationship is described by Michaelis–Menten kinetics, given by the following equation:

$$V = \frac{{V_{{\text{s}}} \left[ S \right]}}{{K_{{\text{m}}} + S}},$$

where *K*_m_ is the Michaelis–Menten constant and *V*_max_ is the maximum reaction rate. The *K*_m_ is calculated using the Lineweaver–Burk plot which presents a linear relationship between 1/*V* and 1/*S*, respectively. The kinetic parameters in this study were determined by varying the H_2_O_2_ substrate concentration for both free and immobilized CAT. The calculated parameters for free and CAT–Gel–Alg are presented in Table 3. The *K*_m_ value (24.15 mM) for CAT–Gel–Alg was higher than that of the free CAT (20.5 mM). The obtained *K*_m_ for CAT–Gel–Alg suggests a decrease in the affinity of the enzyme toward substrate upon entrapment in the hydrogel matrix, i.e., hydrogen peroxide in comparison to free CAT. As presented in Table 3, the *V*_max_ of immobilized CAT (13,900 μmol H_2_O_2_/mg protein min), was slightly lower than that of free CAT (14,700 μmol H_2_O_2_/mg protein min). The reason for the higher *V*_max_ of the free CAT than the immobilized one can be explained based on the existence of diffusional constraints that restrict substrate access to the enzyme upon immobilization into the hydrogel matrix (Abdel-Mageed et al. 2021b). In general, the catalytic efficiency of CAT is preserved at a higher level after immobilization in comparison to the free CAT. Similarly, Başak and Aydemir (2013) reported a higher *K*_m_ value (33.76 mM, 14.28 mM) and a lower *V*_max_ value (141.9 μmol H_2_O_2_/min, 3076.92 μmol H_2_O_2_/min) for immobilized catalase versus free catalase, respectively. Similary, Kaushal et al. (2018) reported a *K*_m_ = 12.5 mM, *V*_max_ = 33,000 μmol H_2_O_2_/mg protein min for free catalase, and *K*_m_ = 25 mM, *V*_max_ = 26,300 μmol H_2_O_2_/mg protein min for immobilized catalase on chitosan.

**Table3:** Kinetic parameters (*K*m, *V*max) and catalytic efficiency of free CAT and immobilized CAT (CAT–Gel–Alg) in hydrogel matrix

Enzyme sample	*K*_m_ (mM)	*V*_max_ (µmol H_2_O_2_ decomposed/min) × 10^4^	Catalytic efficiency (*V*_max_/*K*_m_) mM H_2_O_2_/µmol H_2_O_2_ × 10^4^
Free CAT	20.59	1.47	0.071
CAT–Gel–Alg	24.15	1.39	0.057

Process conditions: temperature 25 °C, pH 7.0, 50 mM phosphate buffer. Variation of the substrate concentration: 4–40 mM H_2_O_2_ and fixed CAT concentration of 0.2 µg/ml

‘

#### Reusability and operational stability

**Fig. 4:**
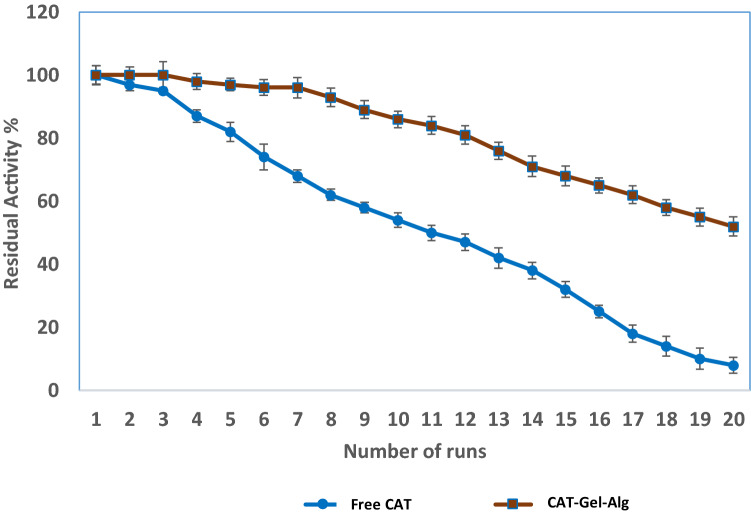
Reusability of free CAT and immobilized CAT (CAT–Gel–Alg) enzyme in consecutive hydrolytic cycles. Residual activity of the immobilized CAT (in %) after 20 cycles of repeated use (initial activity was taken as 100%). CAT activity assay was carried out under standard assay conditions. Each point represents the average of three experiments ± SD

The reuse of immobilized enzymes is an important attribute that is highly significant for industrial applications. Results revealed that CAT–Gel–Alg exhibits superior reusability features, as it retained almost all the original activity for seven consecutive cycles. Afterward, a gradual decrease in activity occurred with 52% residual activity after the 20 consecutive runs of catalytic reactions. In contrast, the relative activity of free CAT gradually decreased until it reached 42% relative activity at run 13 (Fig. 4). The loss of enzymatic activity after repeated use is related to possible conformational changes as a result of consecutive use of the enzyme and the possible poisoning effect of the substrate (Arica et al. 1999). The enzyme was also leached from the immobilization matrix during the washing process. These effects result in alterations in the tertiary structure of the enzyme, which is the primary reason for enzyme deactivation (Abdel-Mageed et al. 2012). It can be concluded that the immobilization procedure used in this study achieved the goal of preserving high enzyme catalytic efficiency for biomedical applications.

#### Storage stability

**Fig. 5:**
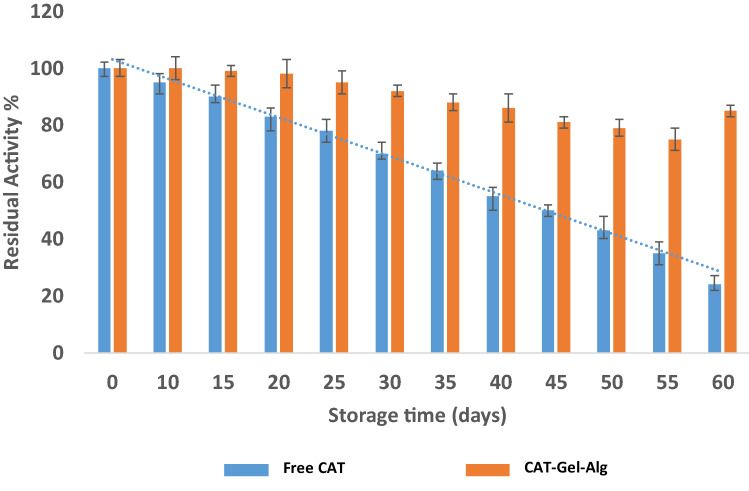
Storage stability of free CAT and immobilized CAT (CAT–Gel–Alg) enzyme for 60 days (50 mM potassium phosphate buffer, pH 7.0, 4 °C). Initial activity at day zero was taken as 100% and CAT activity assay was carried out under standard assay conditions. Each point represents the average of three experiments ± SD

Storage stability is a key factor for the practical application of immobilized enzymes. Free CAT and immobilized Gel–Alg–CAT were stored at 4 °C in potassium phosphate buffer (0.1 mM, pH 7.0) and enzyme activity was carried out at predetermined time intervals for 60 days. Typically, enzymes exhibit short-term stability in the free form. Figure 5 demonstrates that CAT–Gel–Alg activity gradually decreased in comparison to the free form. After 60 days, CAT activity was assayed and 26 and 85% of residual enzyme activity were detected for the free enzyme and immobilized CAT, respectively. These results highlight the superior stability and advantages of immobilized CAT onto hydrogel. The superior stability of CAT upon immobilization has been reported by Kaushal et al. (2018) with 70% residual activity after 60 days’ storage.

### CAT–Gel–Alg efficiency in wound healing

Immediately following burn injury, the inflammatory processes propagated adversely affect the wound healing process. However, following thermal injury, production of short-lived highly reactive oxygen species upsurges in the interstitial fluid with the increase in tissue ischemia, which results in extended cellular damage. Hence, the pathophysiological events following the radical-mediated injury can be avoided via free radical scavengers or inhibitors (Vorauer-Uhl et al. 2002). Given these findings, we investigated the usefulness of CAT administered topically and immediately after thermal burning with hot water. The study was focused on the visible changes implicated by CAT–Gel–Alg administered topically via spreading onto the aggrieved tissues. A scalding burn was elected as a simple procedure to produce uniform consistent burns through hot water exposure. Following the thermal burn induction, all animal groups showed a statistically identical degree of inflammation. In Table 4 the average lesion size areas of each group are presented. 24 h post-trauma, the lesion sizes of the untreated animals (group A) expanded to 155% and of those treated with plain gel (B) to 128% in respect of the initial area (100%). Lesion sizes of the CAT–Gel–Alg treated groups expanded less, 102% in group C. During the first 24 h, group (C) treated with CAT–Gel–Alg exhibited a statistically significant (*p* ≤ 0.05) lower dilatation of lesions in comparison to the untreated animals (A). Additionally, a significant difference (*p* ≤ 0.05) between the group treated with the plain gel (B) and with CAT–Gel–Alg (C) was found. Only a few authors have previously reported the beneficial use of topical free radical scavengers over the systemic ones (Vorauer-Uhl et al. 2001; Abdel-Mageed et al. 2012). After an initial lag time of 1 day, the rate of wound healing was monitored visually by subjective evaluation using two independent inspectors. The inspectors were not informed about the treated group to avoid bias in the assessment. Significant differences in wound healing were observed visually after 48 h for the CAT–Gel–Alg treated groups. Additionally, the treated group showed a reduction in lesion size and an enhanced rate of wound healing after 72 h. It was previously reported that the high water content of hydrogels is perfect for wound hydration and supports conditions in the wound bed for accelerated angiogenesis and even relieves pain (Field and Kerstein 1994). In conclusion, our data suggest that CAT–Gel–Alg is a potent radical scavenger that exhibited a protective effect upon administration in a suitable formulation that is applied immediately after trauma. Whether the use of higher enzyme concentration results in superior edema attenuation needs further investigations.

**Table 4:** The effect of CAT–Gel–Alg treatment on the change in lesion size following burn wound induction

Group	Number	Dosage applied	CAT-activity [%]	Size of lesions		
				Actual size cm^2^	Percentage increase^a^	
Naive (A)	8	No treatment	–	31.65 ± 7.2		155
Control (B)	8	1 gm Plain hydrogel	–	25.67 ± 4.3		128
Treated (C)	8	1 gm CAT–Gel–Alg hydrogel	100	20.45 ± 3.8		102^b^

The mean size of lesions was calculated and results are expressed as average means and standard deviation in cm^2^ at 24 h. Values are means ± SD

^a^The percentage increases were calculated in relation to the initial scalded lesion size of 20 cm^2^

^b^Means are significantly different from native and control at (*p* ≤ 0.05))

## Conclusion and future prospects

This research aimed to synthesize a safe, sustainable, and biocompatible hydrogel of gelatin and sodium alginate with co-incorporation of catalase enzyme for application in wound healing. A simple methodology was employed in this study and was carried out under mild conditions with no high energy input and excluding the use of any organic solvents. In this work, the immobilized CAT exhibited high thermal and storage stability and retained approximately 52% of its relative activity after the 20th catalytic run. Natural gelatin and sodium alginate biopolymers are cost-efficient and proved to offer effective physical immobilization of CAT. Finally, the data obtained in this study suggest a promising effect of topical treatment of thermal injury using antioxidant CAT–Gel–Alg that would offer protection for burned tissues with a promising wound healing process. For future application though, additional investigations are needed to determine the optimal CAT-based hydrogel matrix dosage. It is expected that the outcome of the ongoing and recent advancements in this field will yield a superior and efficient generation of biomaterials with novel possibilities for different biomedical uses.
